# FOSL2 promotes VEGF-independent angiogenesis by transcriptionnally activating Wnt5a in breast cancer-associated fibroblasts

**DOI:** 10.7150/thno.55074

**Published:** 2021-03-05

**Authors:** Xueying Wan, Shengdong Guan, Yixuan Hou, Yilu Qin, Huan Zeng, Liping Yang, Yina Qiao, Shuiqing Liu, Qiao Li, Ting Jin, Yuxiang Qiu, Manran Liu

**Affiliations:** 1 Key Laboratory of Laboratory Medical Diagnostics, Chinese Ministry of Education, Chongqing Medical University, Chongqing 400016, China.; 2 Experimental Teaching Center of Basic Medicine Science, Chongqing Medical University, Chongqing 400016, China.

**Keywords:** cancer-associated fibroblasts, FOSL2, Wnt5a, VEGF-independent angiogenesis

## Abstract

Cancer-associated fibroblasts (CAFs), a predominant component of the tumor microenvironment, contribute to aggressive angiogenesis progression. In clinical practice, traditional anti-angiogenic therapy, mainly anti-VEGF, provides extremely limited beneficial effects to breast cancer. Here, we reveal that FOS-like 2 (FOSL2), a transcription factor in breast CAFs, plays a critical role in VEGF-independent angiogenesis in stromal fibroblasts.

**Methods:** FOSL2 and Wnt5a expression was assessed by qRT-PCR, western blotting and immunohistochemistry in primary and immortalized CAFs and clinical samples. FOSL2- or Wnt5a-silenced CAFs and FOSL2-overexpressing NFs were established to explore their proangiogenic effects. Invasion, tubule formation, three-dimensional sprouting assays, and orthotopic xenografts were conducted as angiogenesis experiments. FZD5/NF-κB/ERK signaling activation was evaluated by western blotting after blocking VEGF/VEGFR with an anti-VEGF antibody and axitinib. Dual luciferase reporter assays and chromatin immunoprecipitation were performed to test the role of FOSL2 in regulating Wnt5a expression, and Wnt5a in the serum of the patients was measured to assess its clinical diagnostic value for breast cancer patients.

**Results:** Enhanced FOSL2 in breast CAFs was significantly associated with angiogenesis and clinical progression in patients. The supernatant from CAFs highly expressing FOSL2 strongly promoted tube formation and sprouting of human umbilical vein endothelial cells (HUVECs) in a VEGF-independent manner and angiogenesis as well as tumor growth *in vivo*. Mechanistically, the enhanced FOSL2 in CAFs was regulated by estrogen/cAMP/PKA signaling. Wnt5a, a direct target of FOSL2, specifically activated FZD5/NF-κB/ERK signaling in HUVECs to promote VEGF-independent angiogenesis. In addition, a high level of Wnt5a was commonly detected in the serum of breast cancer patients and closely correlated with microvessel density in breast tumor tissues, suggesting a promising clinical value of Wnt5a for breast cancer diagnostics.

**Conclusion:** FOSL2/Wnt5a signaling plays an essential role in breast cancer angiogenesis in a VEGF-independent manner, and targeting the FOSL2/Wnt5a signaling axis in CAFs may offer a potential option for antiangiogenesis therapy.

## Introduction

Tumor angiogenesis is a crucial process for the progression and metastasis of solid tumors 1. As primary and metastatic tumors grow, the secretion of a considerable number of unbalanced factors is able to trigger the formation of new vessels through the process of angiogenesis. One of these factors, vascular endothelial growth factor A (VEGFA), is a key mediator of both physiological and pathological angiogenesis and a validated target for antiangiogenesis therapy in the clinic 2. However, extensive clinical experience targeting VEGF-VEGFR agents has shown that this is not always the case 3. For example, several phase III clinical trials failed to provide evidence of overall survival (OS) benefit in bevacizumab arms for patients with metastatic breast cancer. Small molecule antiangiogenic tyrosine kinase inhibitors (TKIs), mainly against VEGFR2, have not shown efficacy in breast cancer treatment until today 4**.** These considerations, combined with the observation that tumor vessels can develop resistance to anti-VEGF therapy 5, highlight the need to identify effective anti-angiogenesis therapies that are based on VEGF-independent targets and can be used in combination with anti-VEGF independent therapy to improve outcomes for patients.

Cancer-associated fibroblasts (CAFs), the major stromal residents and a main producer of paracrine signals, are highly involved in the development of tumors through the secretion of cytokines and angiogenic factors 6. On the one hand, studies have revealed that CAFs may activate multiple signaling pathways in endothelial cells to induce angiogenesis by secreting versatile signaling molecules in addition to VEGFA 7. CAFs promote angiogenesis by recruiting endothelial progenitor cells (EPCs) into invasive human breast cancers, an effect mediated in part by SDF-1 8. Functionally, coculture of FAP-positive CAFs with endothelial cells can stimulate angiogenesis as measured by sprouting in colorectal cancer 9. Interestingly, CAFs from resistant EL4 tumors can also mediate resistance to antiangiogenic therapy via VEGF-independent PDGF-C signaling 10. On the other hand, the tumor stroma exhibits drastic changes at the transcriptional level, which can be useful prognostic predictors of cancer progression. Activation of the pleiotropic transcription factor STAT3 in CAFs promotes colorectal tumor development and correlates with poor prognosis. Strikingly, STAT3 effects in colorectal neoplasia may be at least partially due to modulation of proangiogenic signaling 11. Transcription factor ZEB1 deletion in stromal CAFs reduces the expression and secretion of a variety of paracrine signaling molecules, including FGF2, FGF7, VEGFA and IL6, to suppress tumor initiation, progression and metastasis in a mouse model of breast cancer partially associated with decreased angiogenesis 12. However, the complicated mechanism underlying how breast CAFs promote VEGF-independent angiogenesis in transcriptional addiction is not well understood.

Fos-like antigen 2 (FOSL2/FRA-2) belongs to the activator protein 1 (AP-1) transcription factor family, which includes the various isoforms of Fos and Jun 13. FOSL2 exerts a specific function in bone development 14 and systemic sclerosis (SSc) 15 and appears to have selective physiological and pathological roles in diverse processes, including the development of immune cells 16 and endocrinology processes 17. In addition, enhanced FOSL2 expression has been documented in some carcinomas, such as colon 18, hepatocellular 19, ovarian 20 and breast cancer 21.

Remarkably, FOSL2 transgenic mice resemble the core clinical features of SSc, as they display fibrotic and destructive microvascular manifestations of SSc 22. Increased local FOSL2 levels in cardiac perivascular adipose tissue (C-PVAT) possibly contribute to perivascular angiogenesis, inflammation, and fibrosis 23. AP-1 transcription factors (including FOSL2) act in concert to drive ischemia-induced matrix metalloproteinase-2 (MMP-2) transcription, which plays an essential role in angiogenesis and arteriogenesis 24. However, the role of tumor stroma-derived FOSL2 signaling in tumors has not been clarified.

Wnt5a, a secreted glycoprotein, belongs to the noncanonical Wnt family. Wnt5a and its downstream signaling pathway can regulate fundamental cellular processes, including specification of cell fate, proliferation, and survival. Accumulating evidence indicates that Wnt5a exhibits dual effects on tumor progression 25. For example, Wnt5a plays a pro-tumorigenic role in gastric cancer, non-small cell lung cancer and melanoma, in contrast to thyroid and colorectal cancer 26. However, the role of Wnt5a in breast cancer is controversial and depends on the availability of key receptors and intercellular interactions among different cell types 27. Recently, new evidence has indicated that Wnt5a may play a role in angiogenesis and chemoresistance 28. Our mRNA expression profile suggested high expression of Wnt5a in breast CAFs. The interesting question of whether stromal Wnt5a in breast CAFs is involved in angiogenesis remains to be determined.

Here, we are the first to probe the expression patterns of FOSL2 in breast CAFs and investigate the underlying mechanism, in which FOSL2 is critical for VEGF-independent angiogenesis by transcriptionally regulating Wnt5a. Moreover, we unveiled the clinical significance of the FOSL2/Wnt5a axis to gain insights into its pro-angiogenic function in the development and progression of breast carcinoma, suggesting potential novel therapeutic strategies for anti-angiogenic therapy.

## Material and Methods

### Clinical samples, stromal fibroblast isolation, immortalization, and cell culture

Human breast tumor tissues and their corresponding normal breast tissues (at least 5 cm away from tumor) were obtained from patients at the First Affiliated Hospital of Chongqing Medical University. All the patients involved in this study did not receive any radiotherapy or chemotherapy previously. The experiments were approved by the Ethics Committee of Chongqing Medical University.

The isolation, immortalization, identification and culture of cancer-associated fibroblasts (CAFs) and normal fibroblasts (NFs) from breast tumor patients were previously well established as described in our previous publication 29. NFs and CAFs were cultured in DMEM medium (GIBCO, USA) with 10% FBS (GIBCO, USA) at 37 °C in humidified atmosphere containing 5% CO_2_. HUVECs and MDA-MB-231 cells were cultured in RPMI 1640 medium (GIBCO, USA) containing 10% FBS at 37 °C in humidified atmosphere containing 5% CO_2_.

### RNA interference, plasmids, and reagents

FOSL2- or Wnt5a-knockdown cells were produced by lentivirus-mediated transduction using synthetic short hairpin RNA (shRNA) oligonucleotides (GenePharama, Shanghai, China) according to the manufacturer's protocols. The small interfering RNAs (siRNA) were used to transiently knock down CYP19A1 or FZD5 (GenePharama, Shanghai, China). The sequences of shRNA and small interfering RNA used are listed in Table S1. FOSL2 overexpressing lentivirus were purchased from GeneCopoeia (Guangzhou, China), and viral supernatant was used to infect NFs in the presence of 8 µg/ml polybrene according to the manufacturer's instructions.

The pcDNA3.1(+)-FOSL2 plasmid was created by amplifying FOSL2 from CAFs and was cloned into pcDNA3.1(+) vector. The synthesized nucleotide wild type (WT), truncated or mutant (Mut) Wnt5a promoter was inserted into pGL3 luciferase reporter vector (GenePharma, China), respectively. The reagents used in this study are as follows: the neutralizing antibody against VEGF (Bevacizumab; Roche/Genentech, Switzerland), 0.2 mg/mL; axitinib (Selleck, USA), 5 nM; CCK8 (Beyotime, China), 5 mg/mL; SQ22536 (Selleck, China), 100 µM; H89 (Selleck, China), 5 µM; rWnt5a (R&D Systems, USA), 5 µg/mL; BOX5 (Merck Millipore, USA), 5 µM. The usage of rWnt5a and BOX5 in xenografts was described in animal experiments in detail.

### RNA extraction and qRT-PCR

Total-RNA was extracted using Trizol reagent (Takara, China) and was reverse-transcribed to cDNA using PrimeScript RT reagent kit (Takara, China). Quantitative real-time PCR was performed using SYBR Premix Ex Taq^TM^ II (Takara, China) by CFX96 real-time PCR system (Bio-Rad, USA). The primers used in qRT-PCR are listed in the Table S2.

### Immunohistochemistry (IHC) and immunofluorescence (IF)

The deparaffinized tissue sections at 4 µm thickness were heated for antigen retrieval at 95 °C in citric acid buffer (pH 6.0). After treating with 3% H_2_O_2_, the sections were blocked with 5% goat normal serum, and incubated with primary antibody against CD31 (1:100, ab9498, Abcam, UK) and FOSL2 (1:800, ab124830, Abcam, UK), separately. Microvessel density (MVD) was assessed by CD31 staining as described previously 30. For immunofluorescence, cells were grown on preprepared coverslips for 24 h, then fixed with 4% paraformaldehyde, treated by 0.1% triton-100, and incubated with 5% goat serum. The specific antibodies against α-SMA (1:150, ab5694, Abcam, UK), FAP (1:100, #66562, CST, USA) and CD31 (1:80, ab9498, Abcam, UK) were employed in the IF staining. The normal rabbit IgG was the negative control. The nuclei were stained with DAPI and the images were captured by a fluorescence microscope (Eclipse 80i, Nikon, Japan).

### Luciferase assay

CAFs (1×10^5^ per well) were seeded in 24-well plates and cultured overnight. The cells were transfected with the Wnt5a promoter vectors (pGL3-Wnt5a-WT, pGL3-Wnt5a-truncated, pGL3-Wnt5a-Mut), control vector pGL3 and internal control pRL-TK (Promega, USA) using Lipofectamine 2000. After culture for 30 h, the cells were lysed in passive lysis buffer (Promega, USA), and luciferase activity was measured. Each group was analyzed in triplicate.

### Western blotting and ELISA assays

Cell lysates were separated by 10% SDS-PAGE. The specific primary antibodies used in western blotting analysis were as follows: FOSL2 (1:1000, ab124830, Abcam, UK), Wnt5a (1:1000, ab179824, Abcam, UK), VEGFA (1:1000, ab1316, Abcam, UK); p-VEGFR2 (1:800, D155165, Sangon, China), T-VEGFR2 (1:1000, D151118, Sangon, China); T-AKT (1:1000, #9272, CST, USA), p-AKT (1:1000, #2965, CST, USA), T-ERK (1:1000, #9102, CST, USA), p-ERK (1:1000, #4348, CST, USA), T-P65 (1:1000, #8242, CST, USA), p-P65 (1:1000, #3033, CST, USA) and β-actin (1:1000, Biosharp, China).

For detection of secreted Wnt5a in the supernatant of NFs and CAFs, proteins contained in the supernatant were concentrated using an Amicon Ultra15 Centrifugal Filter Device (Merck Millipore, USA). Concentrated supernatants (30 μL) were used for western blotting analysis. For ELISA, conditioned medium was collected from 1×10^6^ NFs or CAFs in a 6-well plate, and the concentrations of VEGFA (Raybiotech, USA) and cAMP (R&D System, USA) were determined by a standard ELISA Kit according to the manufacturer's instructions. To minimize the effect of cell number on the concentration of secreted proteins, supernatants from fibroblasts seeded at the same cell density were collected after a short incubation period (24 h), and cell numbers were counted at the CM collection time point. For detection of Wnt5a in patient serum and cellular Wnt5a of tumors, patients' peripheral venous blood was collected before surgery, and the blood and tumor tissue lysate were centrifuged at 3000 r/min for 15 min to collect supernatants. The supernatants from serum and extracts of fresh tumor tissues were used to detect Wnt5a levels using an ELISA kit (Wuhan Purity Biotechnology Company, Wuhan, China) according to the manufacturer's instructions.

### Preparation of conditioned medium (CM) and VEGF-neutralizing antibody treatment

The stromal fibroblasts (1×10^6^) were seeded into a 6-well plate in growth medium for 6 h. After removing the growth medium, FBS-free medium (1 mL) was added to further culture the cells for 24 h, and the supernatant was collected as conditioned medium (CM). To exclude the potential side effects of cell proliferation of CAFs and NFs (e.g., the engineered CAFs and NFs, and their control cells) on CM, cell proliferation, and protein concentration (using Bradford assay kit, Beyotime, China) of CM were carefully determined at the CM collection time point. For neutralization experiments, the neutralizing antibody against VEGF was preincubated at 4 °C with 1 mL supernatant for 6 h, and the removal of the VEGF effect in the supernatant was proved by ELISA assay. Then, the supernatant was used for HUVEC coculture in tubulogenesis or sprouting experiments. To rule out protein differences in the potential number of cells at the terminal time point of CM harvest, the protein concentration of CM derived from stromal fibroblasts was normalized using the protein concentration deviation value between the test fibroblasts and their control fibroblasts and adjusted to the corresponding protein concentration of control cells to diminish the personal error of cell planting.

### Invasion assays

Stromal fibroblast-mediated HUVEC invasion was evaluated using an 8 µm pore chamber (Millipore, USA) coated with Matrigel (Corning, USA) as described previously 30. Cells were allowed to invade toward the medium in the lower chamber, and the invaded HUVECs on the opposite side of the filter were stained with 0.5% crystal violet and counted after 16 h.

### Tubule formation and three-dimensional sprouting assays

Growth factor-reduced Matrigel basement membrane matrix (Corning, USA) was slowly thawed on ice, and 50 µL of Matrigel was added to each well of a 96-well plate for polymerization. A total of 1×10^4^ suspended HUVECs were added on top of the Matrigel matrix and treated with the supernatants from CM pretreated with or without VEGF-neutralizing antibody, axitinib, rWnt5a (5 µg/mL) or BOX5 (a Wnt5a antagonist, 5 µM) for 24 h. After incubating at 37 °C for 5 h, pictures were captured in a 40× field and analyzed using ImageJ software. For endothelial cell sprouting assays, HUVECs (1×10^3^) were cultured with medium containing 0.24% carboxymethylcellulose in nonadherent round-bottom 96-well plates overnight. Spheroids were collected by centrifugation at 400 × g and mixed with 2 mg/mL collagen type I (Corning, USA) and FBS-free medium. The three-dimensional fibrin gel was then covered with CM and incubated at 37 °C for at least 24 h. Endothelial sprouting was analyzed with at least 10 spheroids per group and quantified by recording the branch number under a microscope.

### Chromatin immunoprecipitation assay

Chromatin immunoprecipitation (ChIP) assays were performed using the SimpleChIP Plus Enzymatic Chromatin IP Kit (Magnetic Beads) #9005 (CST, USA). Three primer sets were designed to flank the related putative FOSL2-binding sites in the promoter region of VEGF (Table S3). Briefly, FOSL2-transfected NFs (1.2×10^7^) were fixed in 1% paraformaldehyde and sonicated (60 W). Then, the chromatin associated with FOSL2 was pulled down using an anti-FOSL2 antibody or control human IgG. The amounts of the specific DNA fragments were then quantified by real-time polymerase chain reaction (PCR) and normalized to the genomic DNA prepared from the same cells. Each group was analyzed in triplicate.

### E2 production assay

Cells were seeded in six-well plates (2×10^6^ cells/well) and cultured to 50% confluence. Testosterone was added at 100 nM. The cells were cultured for 48 h, and the medium was harvested for E2 detection using the Access E2 Immunoassay System (Beckman Coulter, USA) in the Endocrinology Laboratory, the First Affiliated Hospital of Chongqing Medical University.

### Orthotopic xenografts

The orthotopic tumor xenograft models were generated as described previously 30. Animal experiments were approved by the animal care ethics committees at Chongqing Medical University. MDA-MB-231 cells (1×10^6^) were mixed with an equal number of CAFs/scramble, CAFs/shFOSL2, NFs/vector or NFs/FOSL2 in 200 µL of PBS:Matrigel at a 1:1 ratio and subcutaneously injected into 4-week-old female nude mice (7 mice per group). Tumor size was measured by calipers every 4 days; volume was calculated ((L × W^2^) × 0.5). When the tumor volume was approximately 50 mm^3^, a group of mice implanted with mixtures of MDA-MB-231 and CAF/scramble or CAF/shFOSL2 was separately intraperitoneally administered anti-VEGF antibody (5 mg/kg, twice a week; for 5 consecutive weeks) (Bevacizumab; Roche/Genentech); a group of mice injected with mixtures of MDA-MB-231 and CAF/shFOSL2 was treated with rWNT5A (1 ng/1 g body weight, twice weekly, for 5 consecutive weeks); and a group of tumor burden mice with MDA-MB-231 and NF/FOSL2 was injected with BOX5 (0.5 µg/1 g body weight, once per day, for 5 consecutive weeks). The mice were sacrificed, and xenografts were harvested at 40 days. At the end of animal experiments, tumors were serially sectioned into 4 μm sections and stained with CD31 for subsequent blinded evaluation of MVD.

## Results

### High FOSL2 expression in breast stromal CAFs is closely associated with tumor angiogenesis and clinical progress

**Figure 1:**
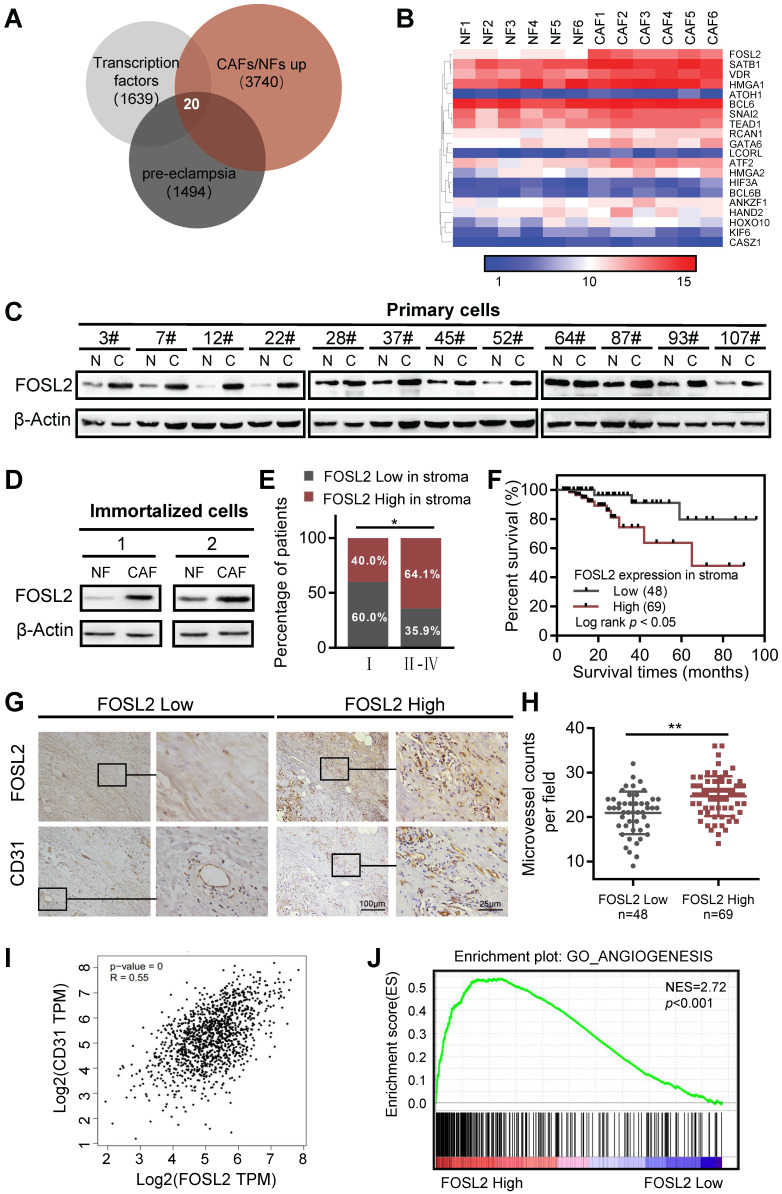
** FOSL2 expression is upregulated and positively correlates with angiogenesis in breast cancer CAFs.** (A) The known human transcription factors were downloaded from a public database (http://humantfs.ccbr.utoronto.ca/), the upregulated genes in CAFs were identified using microarray data, and the downregulated genes of preeclampsia from the GEO database of GSE99007 were analyzed by bioinformatics. The Venn diagram shows twenty dysregulated transcription factors in CAFs. (B) Heatmap of the dysregulated transcription factors in 20 paired breast CAFs and NFs detected by Agilent mRNA microarrays (fold changes>1.5; *p*<0.05, CAFs vs NFs). (C, D) western blot analysis to determine the levels of FOSL2 protein in 12 paired primary CAFs and their NFs isolated from breast tumor tissues (C) and in 2 paired immortalized NFs and CAFs (D). β-actin was the loading control. (E) Percentages of specimens with low or high expression of FOSL2 according to stage. (F) Kaplan-Meier survival curve of overall survival in 117 patients with carcinoma according to stromal FOSL2 expression. (G) Representative IHC staining image showing the increased microvessel density in breast cancer tissues with high FOSL2 expression. (H) Quantity of MVD in breast tumor tissues with high or low FOSL2 expression. (I) The correlation between the levels of FOSL2 and CD31 in breast tumor samples from TCGA (r=0.55, *p*<0.001). (J) GSEA analysis of TCGA database showing a positive correlation between FOSL2 expression levels and the enrichment of angiogenesis-related genes.

Studies investigating the link between CAFs and angiogenesis have been increasingly revealing and demonstrating that the crosstalk between CAFs and endothelial cells facilitates multiple hallmark processes in angiogenesis, where CAFs can even mediate resistance to antiangiogenic therapy. To better understand the characteristics and functions of CAFs associated with tumor angiogenesis, we set out to investigate the transcriptome of breast stromal fibroblasts by microarray analysis using primary CAFs and NFs derived from breast tumor tissues. A set of transcription factors was found to be upregulated in CAFs. Among them, 20 transcription factors were inversely downregulated in preeclampsia (PE) (Figure 1A). The placental phenotype of PE is largely attributed to vascular dysfunction of the placenta, which has been driven by an imbalance of angiogenic signals. PE is concomitant with significantly altered decreased angiogenic factors (VEGF, placental growth factor (PLGF), and angiopoietin-2 (Ang-2)) and increased soluble antiangiogenic factors (e.g., soluble fmslike tyrosine kinase-1 (sFlt-1), and soluble endoglin (sEng)) 31. As shown in the heatmap of the 20 TFs from the mRNA microarray (Figure 1B), FOSL2, SATB1 and GATA6 in CAFs were identified as candidate transcription factors in the progression of angiogenesis in breast cancer. We isolated 12 pairs of primary CAFs and corresponding NFs from breast cancer and noncancer tissues, and the purities of CAFs were identified by the activated fibroblast biomarkers α-SMA, FAP and vascular endothelial marker CD31 (a biomarkers of endothelial cells as control) as previously reported (Figure S1A). After analysis of other conventional CAF marker genes (e.g. S100A4, PDGFRβ, PDPN, and CAV1), S100A4 and CAV1 were found to be increased in primary breast CAFs compared with NFs at the mRNA level (Figure S1B). The expression of three candidate transcription factors was then verified in 3 cases of primary mammary fibroblasts from breast reduction surgery and 12 paired primary breast CAFs and NFs by qRT-PCR, and FOSL2 had the most significant and stable expression in primary CAFs (Figure S1C). Subsequently, the high level of FOSL2 protein was almost confirmed in 12 paired primary CAFs by western blotting analysis (Figure 1C). Likewise, the high level of FOSL2 in immortalized CAFs was also detected by qRT-PCR (Figure S1D) and western blotting (Figure 1D), which had a similar expression pattern as in primary CAFs (thus, the immortalized CAFs could be used as a cell model in this study). To further understand the expression of stromal FOSL2 in breast tumor tissues and evaluate the correlation of stromal FOSL2 with the clinical characteristics of breast cancer, FOSL2 proteins were examined in 117 paraffin-embedded breast tumor tissues by immunohistochemistry. Detailed information on stromal FOSL2 expression and the clinical characteristics of breast cancer patients are listed in Table S3. The high level of stromal FOSL2 was significantly related to tumor stage (Figure S1E and Figure 1E). In addition, Kaplan-Meier survival curves indicated that the expression of stromal FOSL2 was inversely correlated with the overall survival of patients (Figure 1F). Meanwhile, the denser microvessel density (MVD), which was evaluated by CD31 staining, was greater in stromal FOSL2 highly expressed tumor tissues than in stromal FOSL2 low expressed tissues (*p*<0.001) (Figure 1G-H). The correlation between the levels of FOSL2 mRNA and the microvessel biomarker CD31 was discerned from the analysis of bulk data from The Cancer Genome Atlas (TCGA) database, and the results revealed a positive correlation between FOSL2 mRNA expression and CD31 expression (Figure 1I). Using gene set enrichment analysis (GSEA) and Gene Ontology (GO) analysis, we found that the FOSL2-regulated gene signature was positively correlated with the enrichment of angiogenesis-related gene signaling (Figure 1J, and Figure S1F). Collectively, these data indicate that FOSL2 in stromal fibroblasts potentially affects angiogenesis in breast tumors.

### Enhanced FOSL2 in CAFs contributes to breast tumor angiogenesis

**Figure 2:**
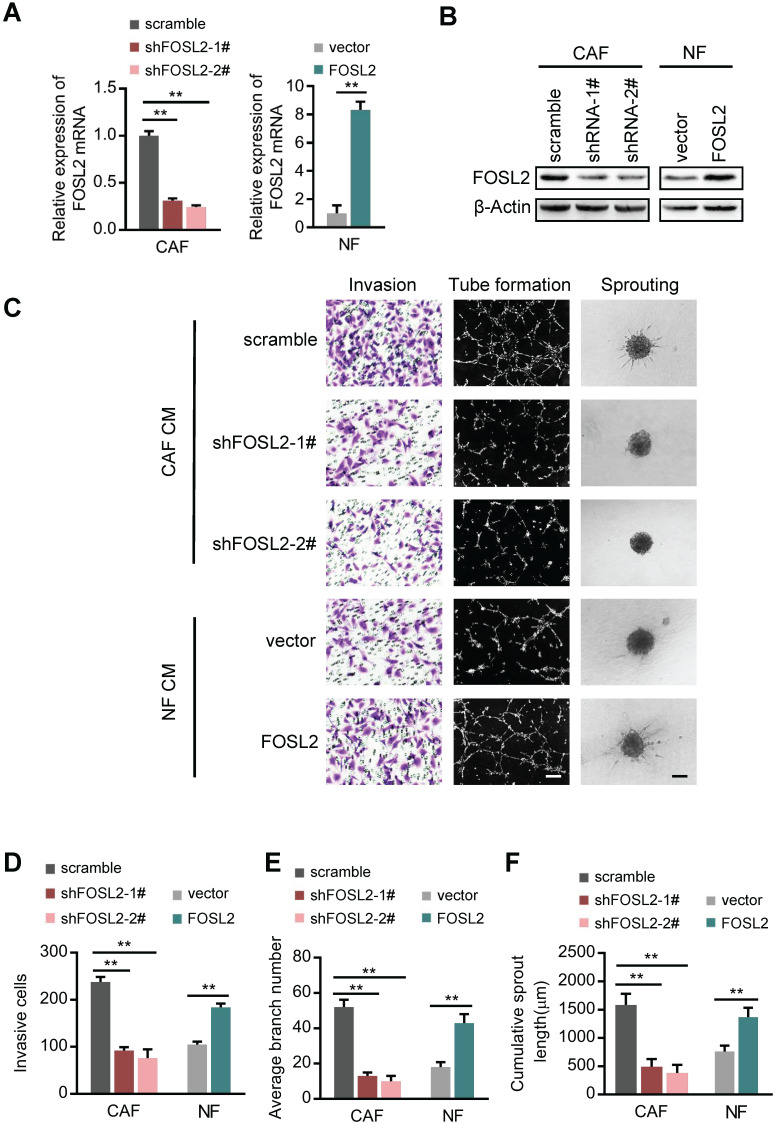
** FOSL2 promotes CAF angiogenesis *in vitro*.** (A-B) FOSL2 levels were detected by qRT-PCR or western blotting in the indicated cells (**p<0.01). (C) Representative images of HUVEC recruitment (left panel), HUVEC tube formation (middle panel), and HUVEC spheroid spouting (right panel) using conditioned media (CMs) are shown (scale bar, 100 μm). (D-F) Quantification analysis of the recruitment of HUVECs (D), the average branch number (E), and the cumulative sprout length (F) of the different groups are shown.

To examine the role of FOSL2 in angiogenic behavior, we successfully established endogenous FOSL2 stably knocked down CAFs (CAFs/shFOSL2) and ectopic FOSL2 stably expressed NFs (NFs/FOSL2), which were confirmed at both the mRNA and protein levels (Figure 2A-B). Ectopic FOSL2 in NFs or FOSL2 silencing in CAFs led to activation of NFs or inactivation of CAFs, as identified by α-SMA (Figure S2A). By employing a coculture system including human umbilical vein endothelial cells (HUVECs) and conditioned medium (CM) derived from CAFs or NFs, we observed a significant decrease in the invasion potential of HUVECs and the cumulative number of tubes in the coculture group treated with supernatant derived from FOSL2-silenced CAFs compared with the group treated with supernatant derived from the control CAFs (Figure 2C, top panels; Figure 2D-E). To better mimic *in vivo* blood vessel formation, we conducted HUVEC spheroid sprouting assays in 3D culture. The results showed that the cumulative sprout length (CSL) was significantly shorter in the system group treated with supernatant from FOSL2-knockdown CAFs than in the control group (Figure 2C, top panels; Figure 2F). In contrast, the invasion, tubule formation and spheroid sprouting abilities of HUVECs were dramatically enhanced by CM from FOSL2-overexpressing NFs (Figure 2C, bottom panels; Figure 2D-F). To adequately understand the pro-angiogenic functions of the CM derived from the engineered CAFs or NFs, we evaluated the different types of vectors on the cell proliferation of CAFs or NFs and the protein concentrations in the medium at the terminal time point of the experiment. The proliferation of CAFs or NFs was not significantly influenced by FOSL2 expression (Figure S2B). The protein concentrations between NFs and CAFs in FBS-free medium or in 10% FBS-containing growth medium also had no significant differences at the terminal time point of CM (Figure S2C-D), indicating that the pro-angiogenic behavior of CM was just induced by FOSL2 levels in CAFs or NFs. Previous studies revealed that FOSL2 was abundantly expressed in breast tumor cells. The effect of CM derived from CAFs or NFs on tumor cells was evaluated. Interestingly, MDA-MB-231 and BT-549 breast cancer cells cocultured with CM from FOSL2-silenced CAFs had reduced proliferation (Figure S2E-F) and invasive ability (Figure S2G-I) in comparison with CM from control CAFs. In contrast, the proliferation (Figure S2E-F) and invasion (Figure S2G-I) abilities of breast tumor cells were increased by CM from FOSL2-overexpressing NFs. Taken together, these results suggest that FOSL2 in breast CAFs plays a positive role in angiogenesis.

### FOSL2 promotes CAF angiogenesis in a VEGF-independent manner

**Figure 3:**
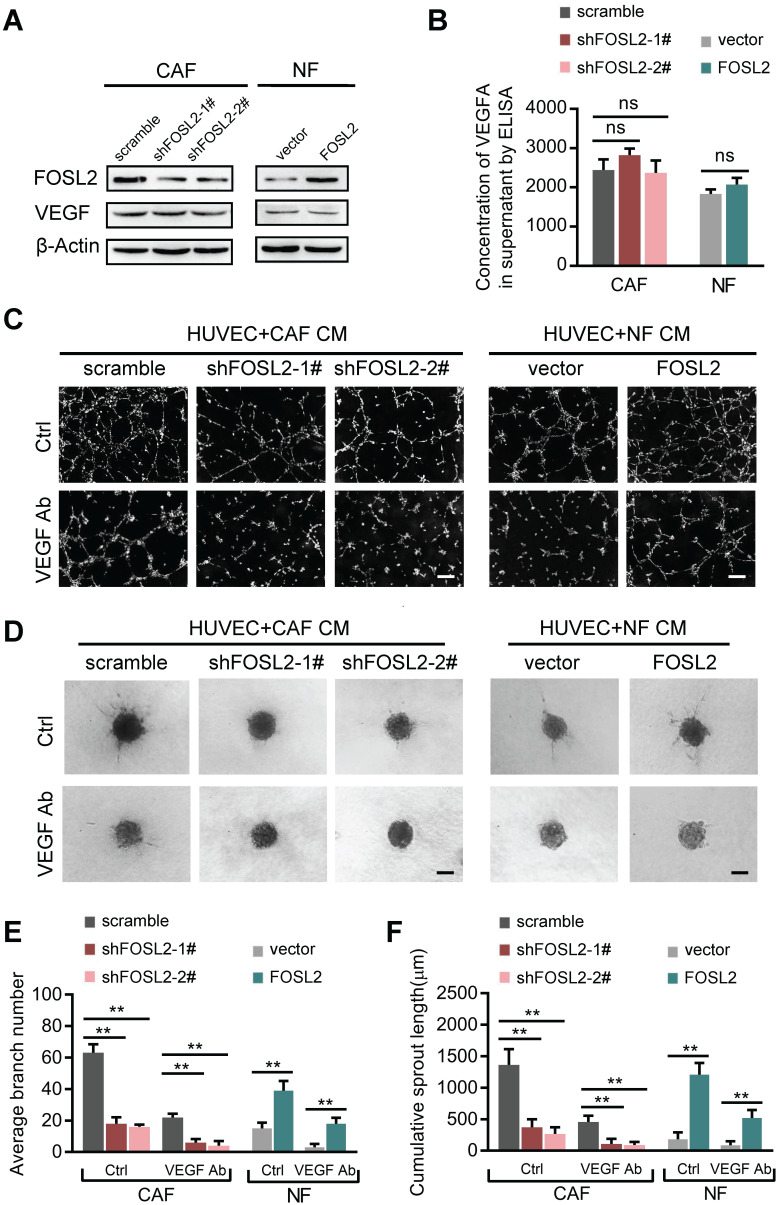
** FOSL2 promotes CAF angiogenesis in a VEGF-independent manner.** (A) VEGFA levels were detected by western blotting and normalized to β-actin in the indicated cells. (B) ELISA was used to determine the VEGFA concentrations in the supernatants of the indicated cells. (C, E) Representative images of the formation of HUVEC tubes following incubation with CM derived from CAFs or NFs treated with VEGF Ab; the average branch number was calculated. (D, F) Representative images of the spheroid spouting of HUVECs following incubation with CM collected from CAFs or NFs treated with VEGF Ab; the cumulative sprout length was calculated (scale bar, 100 μm).

Among the known angiogenic regulators, VEGFA is a key mediator. We thus evaluated whether FOSL2 can regulate VEGFA expression in breast CAFs. Loss of endogenous FOSL2 in CAFs or transduction of ectopic FOSL2 into NFs had almost no effect on VEGF expression (Figure 3A, and Figure S3A). Consistently, FOSL2 expression was found to be extremely limited in association with VEGFA levels analyzed using TCGA database (Figure S3B, r=0.17, *p*<0.001). Concomitantly, knockdown of FOSL2 in CAFs or overexpression of ectopic FOSL2 in NFs had no effect on VEGFA secretion in the supernatants derived from these engineered fibroblasts and the corresponding control cells (Figure 3B). Indeed, CM derived from CAFs or ectopic FOSL2-expressing NFs maintained the ability to promote tubule formation and sprouting of HUVECs in the presence of anti-VEGF antibody (Figure 3C-F) or axitinib (an inhibitor of VEGFR) (Figure S3C-F). These data suggest a VEGF-independent proangiogenic function of FOSL2 in CAFs.

### FOSL2 is regulated by estrogen/cAMP/PKA signaling

**Figure 4:**
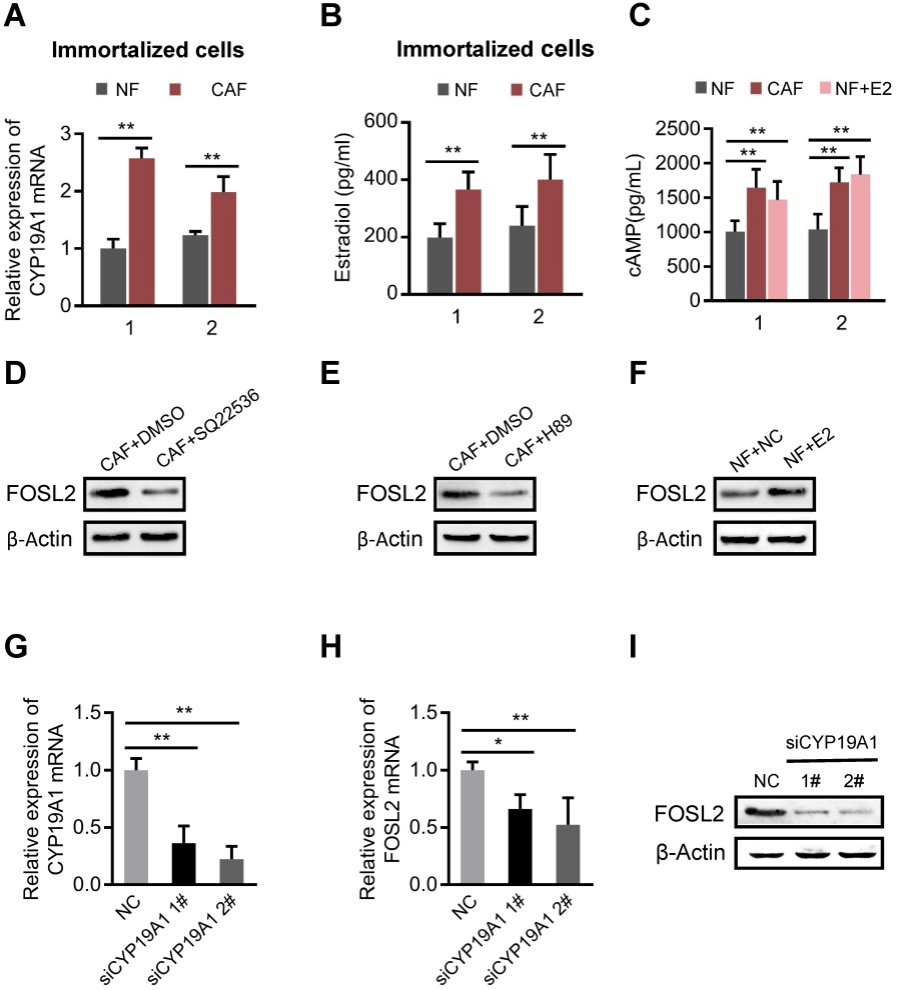
** FOSL2 is regulated by the estrogen/cAMP/PKA signaling axis.** (A) The mRNA levels of CYP19A1 were determined by qRT-PCR in CAFs and NFs. Gene expression was normalized by β-actin (**p<0.01). (.B) Testosterone (100 nM) was added to the culture medium of breast NFs and CAFs. E2 concentrations were determined by chemiluminescence immunoassay (**p<0.01). (C) The production of cAMP in NFs and CAFs was detected by an ELISA Kit (R&D System, USA). Production of cAMP was increased under treatment with E2 (100 nM) in NFs (**p<0.01). (D-F) Protein levels of FOSL2 were detected in CAFs stimulated with SQ22536 (100 μM) (D) and H89 (5 μM) (E) or NFs treated with E2 (100 nM) (F) for 12 h. (G) CYP19A1 levels were detected by qRT-PCR in CAFs transfected with CYP19A1 siRNA (siCYP19A1) and control cells (**p<0.01). (H, I) FOSL2 levels were evaluated by qRT-PCR (H) and western blotting (I) analysis in the indicated cells (*p<0.05, **p<0.01).

The mammary gland is a complex organ that undergoes hormonally regulated changes. CAFs are the major cell type in stroma that converts androgens into estrogen by aromatase, a critical, rate-limiting enzyme in the synthesis of estrogen in the breast cancer microenvironment 32. As expected, the mRNA level of aromatase (CYP19A1) (Figure 4A and Figure S4A) and the amount of synthesized estrogen (E2) were notably higher in CAFs than in NFs (Figure 4B). Our previous study found that E2 could efficiently activate downstream signaling through the cAMP/PKA axis in breast stromal fibroblasts 33. Indeed, more intracellular cAMP production was detected in CAFs or in NFs treated with E2 (Figure 4C). We asked whether FOSL2 expression could be regulated by the estrogen/cAMP/PKA signaling pathway in CAFs. Astoundingly, the increased FOSL2 levels in immortalized or primary CAFs were notably reduced by SQ22536 (a cAMP inhibitor) (Figure 4D, and Figure S4B) and H89 (a PKA inhibitor) (Figure 4E, and Figure S4C); conversely, a higher level of FOSL2 was detected in immortalized or primary NFs stimulated by E2 (Figure 4F, and Figure S4D). Furthermore, efficient knockdown of CYP19A1 in CAFs (Figure 4G) led to decreased FOSL2 (Figure 4H-I). Taken together, these data demonstrate that the local estrogen/cAMP/PKA signaling pathway in breast CAFs is involved in regulating FOSL2 expression.

### FOSL2 transcriptionally activates Wnt5a and leads to increased Wnt5a secretion from CAFs

**Figure 5:**
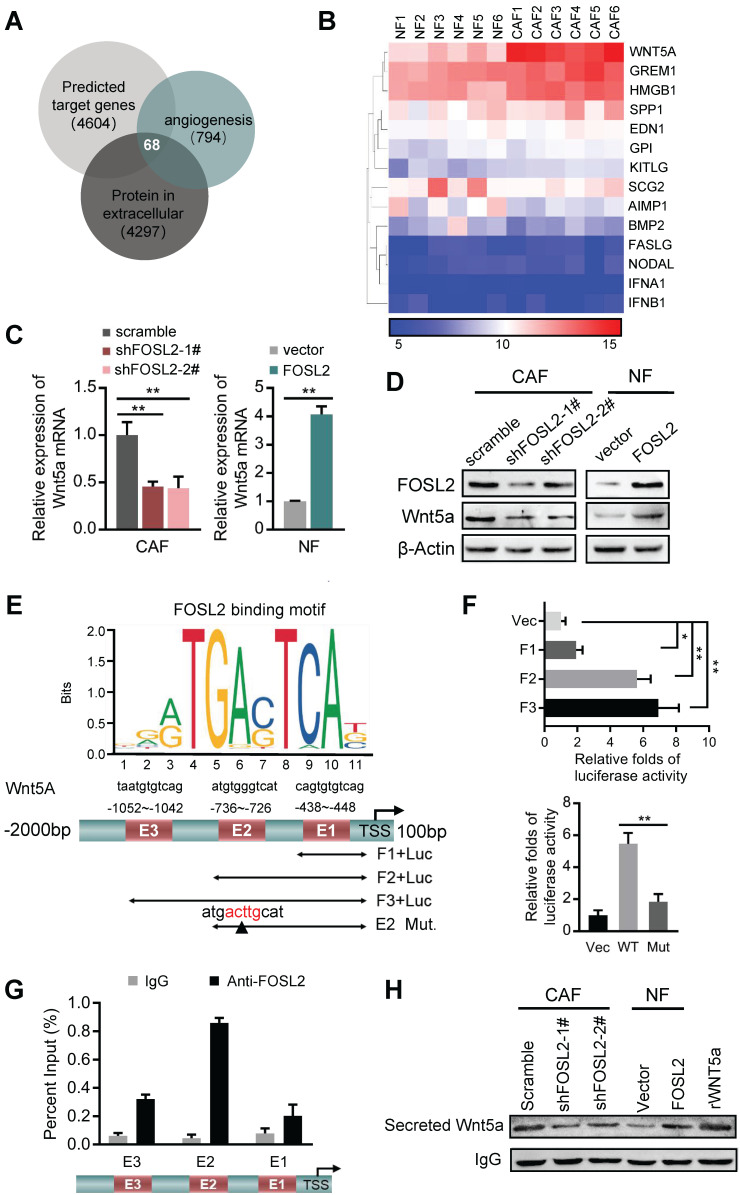
** FOSL2 transcriptionally activates Wnt5a.** (A) The predicted target genes regulated by FOSL2 from the Cistrome database and a set of genes related to angiogenesis and the reported proteins in the extracellular matrix downloaded from AmiGO Gene Ontology were used in our analysis to acquire the FOSL2-regulated angiogenesis genes. Sixty-eight putative FOSL2 targets are shown. (B) The significantly altered cytokine genes (fold changes>1.5 times, CAFs vs NFs) were identified by microarray analysis for primary CAFs and NFs. (C-D) Expression of Wnt5a was determined by qRT-PCR (C) or western blotting (D) in the indicated stromal fibroblasts (**p<0.01). (E) Schematic diagram of canonical FOSL2-binding motif (JASPAR Database) and potential FOSL2 responsive elements (E1, E2, E3) in the Wnt5a promoter. Full-length and truncated Wnt5a promoter or E2 mutated promoter are shown. (F) Transcriptional activities of FOSL2 on Wnt5a were determined by luciferase reporter assay as CAFs were transfected with full-length or truncated Wnt5a promoter (upper panel) and E2 wild-type or mutated reporter plasmids (down panel). (G) ChIP assays were performed to analyze FOSL2 binding to the Wnt5a promoter using an anti-FOSL2 antibody. The E2 site was significantly enriched in comparison with the E1 or E3 site. (H) western blotting to determine Wnt5a collected from CM derived from the indicated cells.

To further explore the functions of stromal FOSL2 in regulating angiogenesis, we identified target genes of FOSL2 by merging the data acquired from targets predicted by the Cistrome database (http://cistrome.org/db/) with the potential genes associated with angiogenesis and secretion of extracellular proteins from the AmiGO database (http://amigo.geneontology.org/). Sixty-eight candidates that may be regulated by FOSL2 and related to angiogenesis were identified (Figure 5A). After comparison with our mRNA expression profile of CAFs/NFs, 14 candidate genes were aberrantly expressed in breast CAFs (Figure 5B). Using a protein network analysis, we found that Wnt5a was in the core of the network among these potential transcriptional targets of FOSL2, indicating that Wnt5a is a major candidate target of FOSL2 (Figure S5A). In accordance with this finding and our previous RNA-sequencing data of CAFs/NFs, Wnt5a was markedly upregulated in breast tumor tissues compared with adjacent nontumor tissues using a public database including 1085 cases of tumors and 291 cases of normal tissues (Figure S5B), suggesting increased Wnt5a in the tumor stroma. Further analysis proved that Wnt5a was significantly changed at the mRNA and protein levels in FOSL2-knockdown CAFs or FOSL2-overexpressing NFs (Figure 5C-D). To further understand whether Wnt5a is a direct transcriptional target of FOSL2, a bioinformatics analysis and sets of experiments were carried out. Bioinformatics analysis showed that there were three potential FOSL2 binding consensus sequences (E1, E2 and E3) in the Wnt5a promoter (Figure 5E). Checked by luciferase reporter assay, we found that the reporter containing E2 (Figure 5E) had high transcriptional activity (Figure 5F, upper panel), and mutation of the E2 motif (Figure 5E) notably decreased luciferase activity (Figure 5F, down panel), which was further confirmed using chromatin immunoprecipitation assay (Figure 5G), suggesting that consensus E2 is essential for FOSL2-mediated transcriptional activity of Wnt5a. Consistently, more Wnt5a-secreting CAFs were detected than NFs, and the concentration of Wnt5a secreted in the supernatant was significantly reduced in FOSL2-knockdown CAFs compared with that in control CAFs. Conversely, there was increased Wnt5a secretion in FOSL2-overexpressing NFs compared with that in control NFs (Figure 5H). These data demonstrate that FOSL2 can directly regulate Wnt5a expression.

### Wnt5a plays an essential role in the FOSL2-mediated promotion of angiogenesis in CAFs

**Figure 6:**
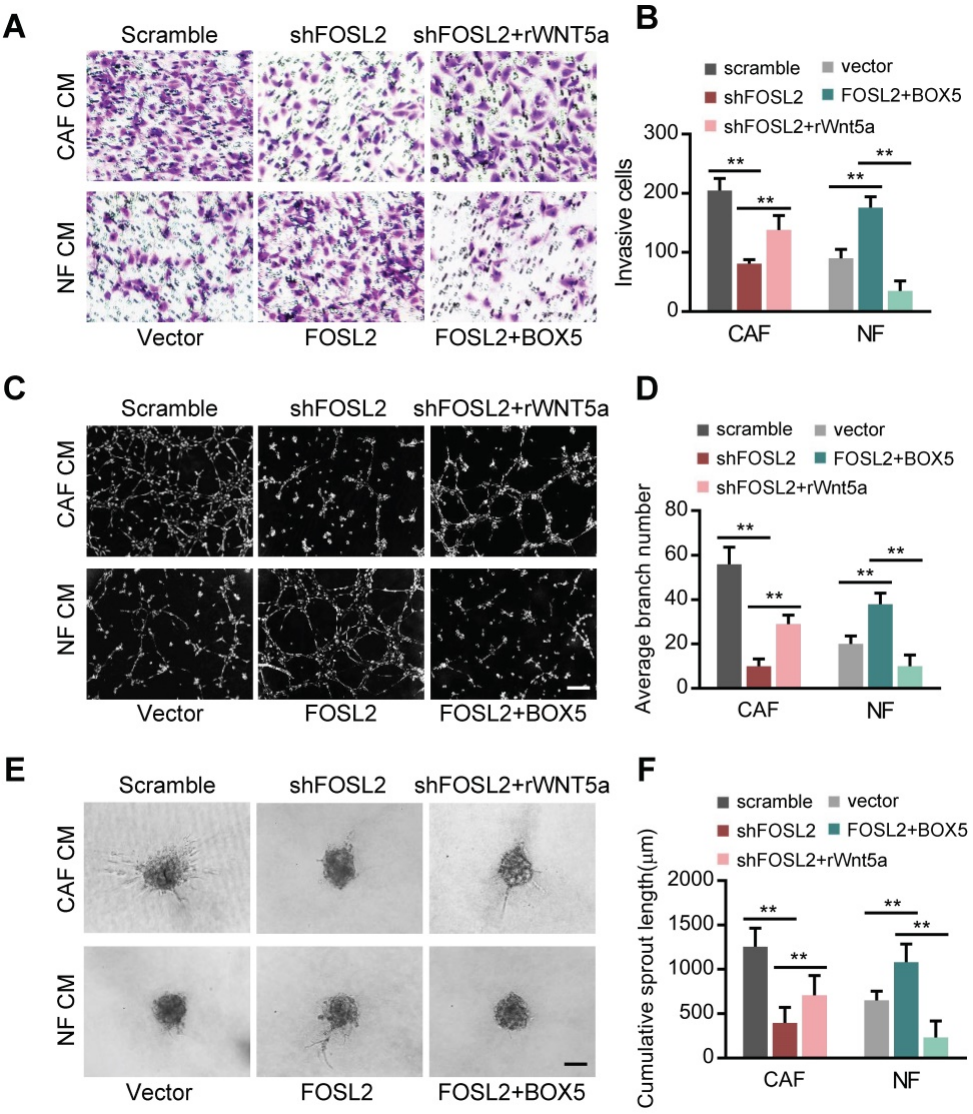
** Wnt5a is involved in the FOSL2-mediated promotion of angiogenesis in CAFs.** (A-F) Cell invasion (A), tubule formation (C), and spheroid spouting (E) abilities of HUVECs were tested in the presence of CM from FOSL2-silenced CAFs treated with or without rWnt5a and the control CAFs or CM from NFs transfected with FOSL2 under treatment with or without BOX5 and their control NFs (scale bar, 40 μm). Quantification analysis of the recruitment of HUVECs (B), the average branch number (D), and the cumulative sprout length (F) of the different groups are shown.

To investigate the pro-angiogenic role of Wnt5a in breast CAFs, we transfected a retrovirus-mediated shRNA specifically against Wnt5a in CAFs and an ectopic Wnt5a construct into NFs. Wnt5a overexpression in NFs (NF/Wnt5a) or loss of endogenous Wnt5a in CAFs (CAFs/shWnt5a) resulted in marked changes in the invasion, tubule formation and spheroid sprouting abilities of HUVECs (Figure S5C), indicating that Wnt5a, as a downstream target of FOSL2, is essential for CAF-promoting angiogenesis function. Next, we examined the Wnt5a-dependent angiogenesis role of FOSL2 in CAFs. Treatment of HUVECs with supernatant derived from FOSL2-silenced CAFs reduced the cell invasion (Figure 6A, upper panels; Figure 6B, left panel), tube formation (Figure 6C, upper panels; Figure 6D, left panel) and spheroid sprouting (Figure 6E, upper panels; Figure 6F, left panel) potential of HUVECs. The addition of rWnt5a into the supernatant from FOSL2-silenced CAFs rescued the cell invasion, tube formation and spheroid sprouting potential of HUVECs, which resulted from the loss of FOSL2 in CAFs (Figure 6A-F). In contrast, the promoting effects on cell invasion, tubule formation and spheroid sprouting of HUVECs were observed in CM derived from ectopic FOSL2 NFs and mitigated by treatment of the CM with BOX5, a Wnt5a antagonist (Figure 6A, 6C and 6E, down panels; Figure 6B, 6D and 6F, right panels). Thus, this evidence supports the Wnt5a-dependent angiogenesis role of stromal FOSL2 in breast cancer.

### Wnt5a derived from CAFs activates FZD5/NF-κB/ERK signaling in endothelial cells

**Figure 7:**
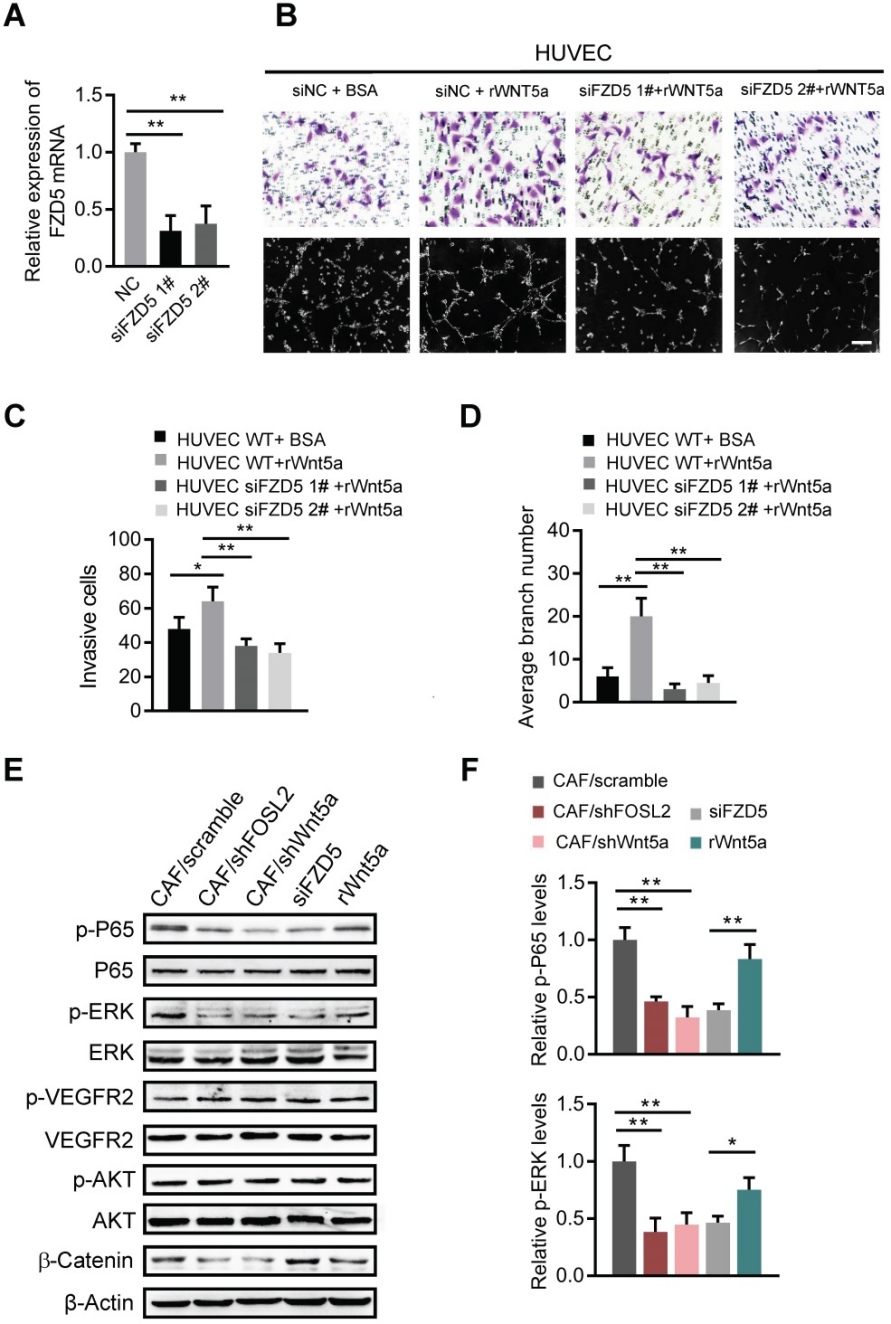
** Wnt5a derived from CAFs activates FZD5/NF-κB/ERK signaling in endothelial cells.** (A) FZD5 levels were detected by qRT-PCR in HUVECs transfected with FZD5 siRNA (siFZD5) and control cells (**p<0.01). (B) Cell invasion and tubule formation of the indicated HUVECs (control HUVECs, HUVECs treated with rWnt5a, and FZD5-silenced HUVECs under treatment with rWnt5a) were assessed (scale bar, 100 μm). (C-D) Quantification analysis of the recruitment of HUVECs (C) and the average branch number (D) of the different groups of HUVECs are shown. (E-F) HUVECs were treated with different CM. The CM was as follows: CAF CM of scramble and loss of FOSL2 or Wnt5a in CAFs, CAF CM with siFZD5, and FBS-free medium with rWnt5a. The total and phosphorylated proteins of P65/NF-κB, ERK, VEGFR2, PI3K/AKT and β-catenin in HUVECs were determined by western blotting. β-actin was used as a loading control. Levels of p-P65 and p-ERK (F) were quantified as relative pixel intensity to β-actin.

Wnt5a can induce signal transduction in target cells by binding to its receptors, the Frizzled family (FZDs), a cysteine-rich seven transmembrane receptor family 25. We asked whether CAF-derived Wnt5a could bind to FZD5, a potential member of the Frizzled family in HUVECs, and stimulate possible downstream signaling pathways. Efficient knockdown of FZD5 in HUVECs (Figure 7A) abolished the cell invasion and tubule formation abilities of HUVECs even in the presence of rWnt5a (Figure 7B-D), suggesting that FZD5 is necessary for Wnt5a in angiogenesis. Several signaling pathways, such as NF-κB, ERK and AKT, are potential downstream signaling pathways of FZDs; thus, the activation of NF-κB, ERK and AKT signaling in HUVECs was examined. As shown in Figure 7E, loss of FOSL2 or Wnt5a in CAFs significantly quenched the activation of NF-κB and ERK in HUVECs stimulated by CAF CM. Similarly, loss of FZD5 in HUVECs rendered NF-κB and ERK signaling unable to respond to CAF CM, and addition of rWnt5a in FBS-free medium directly activated these signaling pathways in HUVECs (Figure 7E-F). However, the activation of VEGFR2 was not affected by silencing FOSL2, Wnt5a, or FZD5 or by exogenous supplementation with rWnt5a. Meanwhile, Wnt5a, as a representative member of the noncanonical Wnt proteins, has been reported to play dual roles in Wnt/β-catenin signaling 34. We found that Wnt5a could stimulate the accumulation of β-catenin in HUVECs (Figure 7F) and thereby promote angiogenesis. These data demonstrate that the Wnt5a/FZD5 axis stimulates the activation of NF-κB and ERK signaling in HUVECs.

### CAFs promote breast tumor angiogenesis in a VEGF-independent manner to fuel breast tumor growth *in vivo*

**Figure 8:**
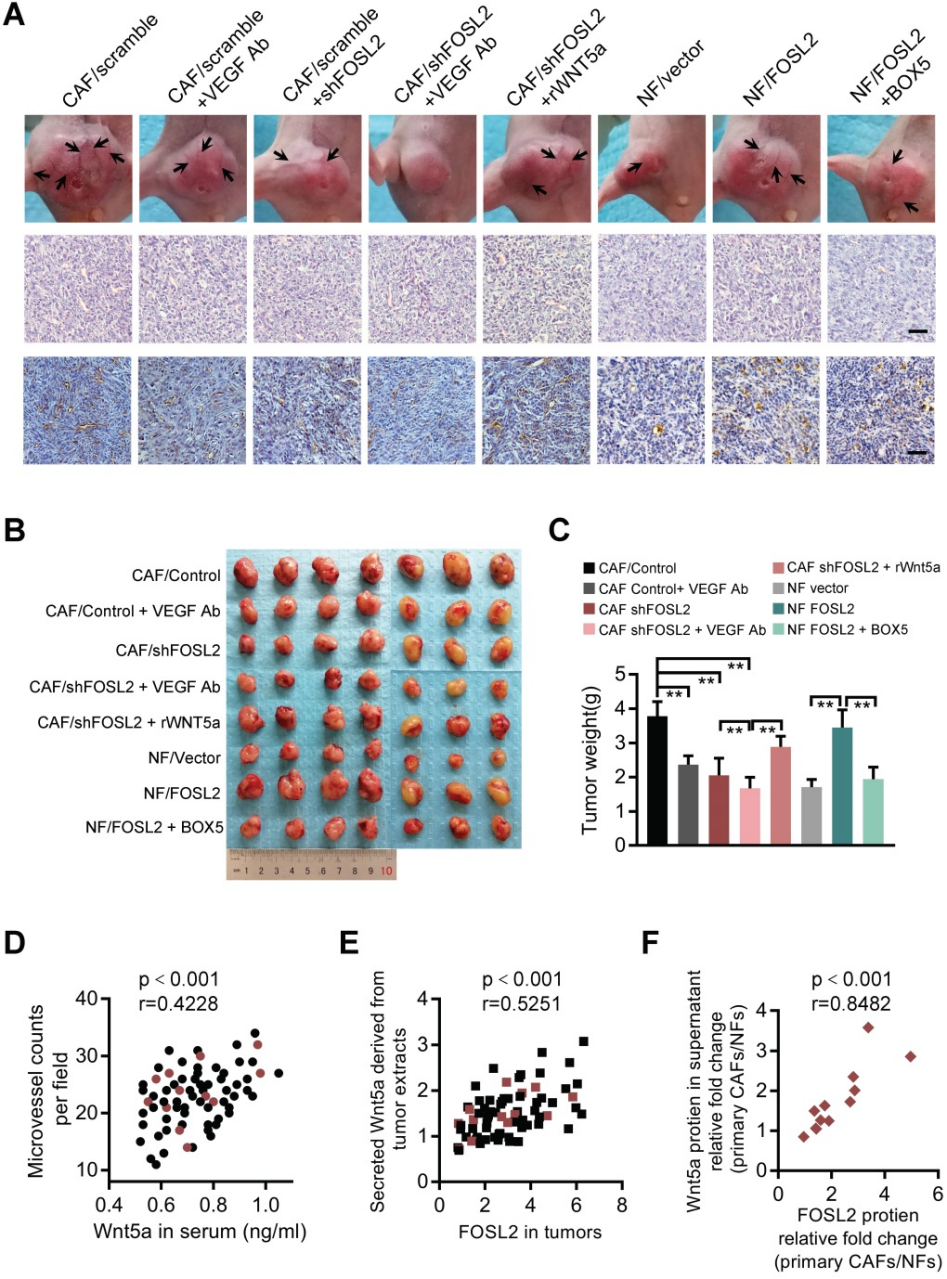
** FOSL2 modulates breast tumor angiogenesis and tumor growth in xenograft mouse models and clinical breast cancer patients.** (A) Representative images of blood vessels on the tumor surface (labeled with black arrow) of mice injected with the mixture of MDA-MB-231 and indicated stromal fibroblasts under treatment with or without VEGF Ab, rWnt5a or BOX5. The blood vessel structures in the H&E and IHC staining of CD31 in tumors of each group of mice are shown (scale bar, 50 μm). (B-C) Representative tumor size (B) and tumor weight (C) for each group of xenograft mice (7 mice in each group) are shown (**p<0.01). (D) The levels of Wnt5a in the serum of 71 patients were positively correlated with the microvascular density in the tumor tissues of breast cancer patients. The red circles represent patients whose primary CAF/NF counterparts were successfully isolated from the tissue. (E) FOSL2 protein levels in breast tumor tissues were positively correlated with Wnt5a in tumor tissue extracts. The red squares represent patients whose primary CAFs/NFs were successfully isolated from the tissue. (F) FOSL2 protein levels were closely correlated with Wnt5a in the supernatant of 12 pairs of primary CAFs and NFs.

To further investigate the proangiogenesis function of the FOSL2/Wnt5a signaling axis *in vivo*, a mixture of the indicated cells was subcutaneously injected into nude mice. Compared with the tumor burden mice injected with MDA-MB-231 cells and CAFs, the tumor burden mice injected with MDA-MB-231 cells mixed with FOSL2-silenced CAFs had fewer blood vessels on the tumor surface and less CD31 staining in tumor tissue; the tumor burden mice injected with a mixture of MDA-MB-231 and FOSL2-overexpressing NFs had more blood vessels and a large tumor size (Figure 8A and Figure S6A). To confirm the number differences of blood vessels attributing to angiogenesis, the mRNA expressions of vascular endothelial (VE)-cadherin (CDH5), a strictly endothelial marker 35, and prosperohomeobox protein1 (prox1), a biomarker for lymphatic endothelial cells 36 were detected in the tumor samples (Figure S6B). The high level of VE-cadherin closely related with microvessel density based on CD31 staining rather than prox1 expression. In contrast to blockage of VEGF signaling alone by anti-VEGF antibody, FOSL2 knockdown in CAFs combined with VEGF signaling inhibitors could augment the suppressive efficiencies to tumor angiogenesis, indicating that FOSL2 acted as an obviously proangiogenic effector in the VEGF-independent pathway. Consistently, treating mice with Wnt5a, the critical pro-angiogenic factor regulated by FOSL2, could partly rescue blood vessels and tumor growth of tumor burden mice injected with FOSL2-silenced CAFs (labeled CAF/shFOSL2 + rWnt5a) in comparison to their control tumors (labeled CAF/shFOSL2). The tumor burden mice injected with NFs had fewer blood vessels and small tumors, and ectopic FOSL2 in NFs could significantly promote blood vessels and tumor growth. However, treatment of tumor burden mice injected with FOSL2-overexpressing NFs using BOX5 (Wnt5a antagonist) mitigated FOSL2-stimulated blood vessels and tumor growth (Figure 8B-C and Figure S6C). In addition, we found that knockdown of FOSL2 in CAFs could increase the sensitivity of tumor cells to VEGF inhibitors *in vivo* (Figure 8A-C, Figure S6A-C). Similarly, loss of Wnt5a, the direct target of FOSL2, in CAFs significantly decreased tumor blood vessel formation and tumor growth (Figure S6D-F). To further evaluate the clinical value of FOSL2-regulated Wnt5a involved in angiogenesis, we detected secreted Wnt5a levels in serum from 71 breast cancer patients and Wnt5a levels in corresponding tumor tissues or secreted Wnt5a in the supernatant of primary stromal fibroblasts isolated from 12 pairs of nontumor and tumor samples and the corresponding FOSL2 expression and vessel density of tumors. High levels of secreted Wnt5a were detected in patient serum, and enhanced serum Wnt5a was positively correlated with microvessel density in breast tumors (Figure 8D). The FOSL2 protein levels in tumor tissues were closely related to the Wnt5a concentration in tumor extracts (Figure 8E). Moreover, the FOSL2 levels in primary stromal CAFs were much higher than those in the corresponding primary NFs, and the enhanced stromal FOSL2 was almost correlated with a high concentration of secreted Wnt5a in the supernatant of CAFs (Figure 8F and S6G), confirming that FOSL2-regulated Wnt5a in stromal CAFs was close to clinical angiogenesis. Collectively, the current study reveals a novel function of breast CAFs. The increased FOSL2 in CAFs plays a pivotal role in promoting angiogenesis by regulating Wnt5a, thus stimulating activation of the downstream FZD5/NF-κB/ERK signaling axis in vascular endothelial cells to fuel tumor angiogenesis VEGF-independent patterns.

## Discussion

The identification of angiogenesis as a prerequisite for the outgrowth of solid tumors boosts the field of angiogenesis research. However, enthusiasm for angiostatic therapy has been hampered due to the limited beneficial effect of these anti-angiogenesis agents on patient survival, especially in breast cancers. The tumor microenvironment (TME) has been considered to play an important role in tumor angiogenesis. In particular, CAFs are gradually viewed as the chief architects of the TME due to their multiple functions, including tumor angiogenesis with intimate crosstalk between endothelial cells 37. In this study, we identified that the transcriptional factor FOSL2 may act as a novel tumor angiogenesis target in breast CAFs. The enhanced FOSL2 in breast CAFs was induced by endogenous aromatase through the E2/cAMP/PKA pathway. Furthermore, the activated CAFs induced by FOSL2 could not promote VEGF but could promote Wnt5a expression, which stimulated angiogenesis through FZD5/NF-κB/ERK signaling, even under the blockage of classical VEGF signaling by anti-VEGF antibody and axitinib. Collectively, the current study suggests that targeting FOSL2 in stromal CAFs may be an effective alternative to overcome tumor refractoriness to anti-VEGF signaling treatment.

Transcription factors (TFs) coordinate the expression of target genes typically through cis-regulatory DNA elements. A small set of lineage-specific master TFs dictate the core transcriptional programs governing cell identity and malignant state. Elucidating the core transcriptional regulatory mechanisms is necessary to understand the fundamentals of molecular carcinogenesis 38. A more recent study has revealed that stomach and intestinal lineage-specific programs are reactivated in Sox2^high^/Sox9^high^ and Cdx2^high^ cancers by comparing the gastrointestinal lineage-specific transcriptome to human gastrointestinal cancer data, which highlights the importance of developmental lineage programs reactivated by gastrointestinal TFs in cancer 39. Strikingly, a novel study has determined that TFs of the FOS family are significantly variable for single E18 fetal mouse mammary cells when compared with the permutated background, suggesting that these factors may contribute to cellular differentiation programs 40. Herein, we found that FOSL2 was upregulated in activated CAFs of breast cancers. The links between FOSL2-mediated breast development and breast tumor angiogenesis suggest that targeting FOSL2 in stromal fibroblasts may have significant value for breast cancer therapy. Meanwhile, we found that FOSL2 was downregulated in preeclampsia (PE) by analyzing the transcriptome of PE, which was ascribed to decreased angiogenesis signaling. These works support our findings that FOSL2 in breast CAFs plays a crucial role in angiogenesis.

The role of CAFs in tumor angiogenesis architecture is increasingly dominant in breast tissues. The focus of our study on breast CAFs in angiogenesis is promising. Recently, in an MMTV-PyMT mouse model of breast cancer, the issue of heterogeneity of CAFs was distinguished by the global gene expression profile using single-cell RNA sequencing. Of note, there are three subtypes of mouse CAFs, including vascular CAFs (vCAFs), matrix CAFs (mCAFs) and cycling CAFs (cCAFs), that are significantly enriched by gene ontology (GO) sets. The vCAF signatures, close to vascular development and angiogenesis, are highly conserved in patient samples of breast tumors 41. Therefore, antagonizing CAFs as an antiangiogenic treatment modality for breast cancer opens the possibility for biomarker-driven development of drugs for precision therapy.

Stromal CAF populations, which engage in pleiotropic processes in tumor angiogenesis, are pivotal to the resistance of VEGF blockade. It has been shown that resistance to antiangiotherapy is partly involved in angio-stimulatory growth factor redundancy and multiplicity. In a seminal paper, compression-induced expression of glycolysis genes in CAFs correlates with angiogenesis gene expression in breast cancer 42. Tumor-induced compression might inevitably be a sustained driver for angiogenesis-related gene expression to cause the redundancy of angiogenesis growth factors. Meanwhile, tumor angiogenesis can become VEGF independent at a more advanced stage because of the production of other proangiogenesis molecules and thus respond poorly to VEGF blockade 43. The release of multiple angiogenesis factors, such as SDF-1 and PDGF-C, by CAFs contributes to resistance 8, 10. Our studies support that, likewise, although VEGFA is considerably expressed in breast CAFs, FOSL2 is one of the key players in promoting angiogenesis in a VEGF-independent manner. FOSL2 in stromal fibroblasts promotes angiogenesis by inducing Wnt5a transcription.

Aberrant Wnt5a signaling is an important event in tumor progression, with oncogenic or tumor-suppressing effects dependent on tumor types 44. In breast cancer, the respectable evidence supports that Wnt5a has oncogenic activity. For example, elevated Wnt5a was detected in basal-like breast cancer cells at both the mRNA and protein levels, and loss of Wnt5a could efficiently inhibit tumor growth in SUM1315 cells *in vivo* 45. In addition, GLIS1, a novel hypoxia-inducible transcription factor, was reported to increase the migration and invasion capacities of breast cancer cells by upregulating Wnt5a, and Wnt5a levels were associated with poorer prognosis in breast cancer patients 46. Notably, several studies reported that Wnt5a was highly expressed in breast stromal cells and associated with poor prognosis in breast cancer. For example, enhanced Wnt5a was proven in breast tumor-associated macrophages (TAMs), which induced invasiveness of breast cancer cells and production of matrix metalloproteinase-7 (MMP-7) and tumor necrosis factor-α (TNF-α) in macrophages 47. A whole-transcriptome analysis of selectively retrieved engulfing breast cancer cells identified a gene signature of mesenchymal stem/stromal cell (MSC) engulfment consisting of Wnt5a, which enhanced distant metastasis 48. Another study revealed that Wnt5a in breast CAFs was significantly downregulated after 1,25D (1alpha, 25 dihydroxyvitamin D3) treatment 49, in which 1,25D elicited obvious tumor suppressive effects. In addition, the role of Wnt5a is emerging in the promotion of proinflammatory and immunosuppressive effects in the tumor microenvironment 50. Furthermore, Wnt5a signaling has been found to be required for the pathological angiogenesis. In a study, the reduced vascularization of matrigel plugs in conditional deletion of the Wnt secretion factor Evi in mouse ECs (Evi-ECKO) could be rescued by introduction of non-canonical Wnt5a 51. The release of Wnt5a from infiltrating monocytes is especially important to drive angiogenesis in inflamed vessels 52. In addition, Wnt5a has been disclosed to play essential role in tumor angiogenesis, such as melanoma 53 and glioblastoma 54. However, whether Wnt5a derived from stromal cell acting a role in angiogenesis has poor understanding. In one research, CD163 positive tumor-associated macrophages (TAM) may induce angiogenesis via crosstalk with malignant epithelial cells to promote Wnt5a expression 55. Here, we confirmed that the increased stromal Wnt5a plays a crucial role in VEGF-independent angiogenesis in breast cancer. Our findings provide important evidence for its oncogenic role in the breast tumor microenvironment, which suggests that targeting Wnt5a in the breast TME may provide a potential option for antiangiogenesis therapy in breast cancer.

Together, our study supports the notion that stromal CAFs promote VEGF-independent pro-angiogenesis processes in breast cancer, and FOSL2-mediated Wnt5a expression and activation of downstream signaling are crucial for VEGF-independent angiogenesis of breast CAFs. The elucidation of reciprocal interactions of breast CAFs with endothelial cells may inspire the development of conceptually novel targeted therapeutics with the aim of thwarting the FOSL2/Wnt5a axis of breast tumor angiogenesis.

## Supplementary Material

Supplementary figures and tables.
