# FOSL2 promotes VEGF-independent angiogenesis by transcriptionnally activating Wnt5a in breast cancer-associated fibroblasts: Erratum

**DOI:** 10.7150/thno.77019

**Published:** 2022-08-18

**Authors:** Xueying Wan, Shengdong Guan, Yixuan Hou, Yilu Qin, Huan Zeng, Liping Yang, Yina Qiao, Shuiqing Liu, Qiao Li, Ting Jin, Yuxiang Qiu, Manran Liu

**Affiliations:** 1 Key Laboratory of Laboratory Medical Diagnostics, Chinese Ministry of Education, Chongqing Medical University, Chongqing 400016, China.; 2 Experimental Teaching Center of Basic Medicine Science, Chongqing Medical University, Chongqing 400016, China.

The authors regret that the original article contains unintentional misuse of tubule formation images in Figure 6C and Figure 7B during figure assembly. The correct versions of Figure 6C and Figure 7B are shown here below. The replacement does not alter result interpretation and conclusion. The authors are deeply sorry and sincerely apologize for any inconvenience or misunderstanding that may have caused.

## Figures and Tables

**Figure 1:**
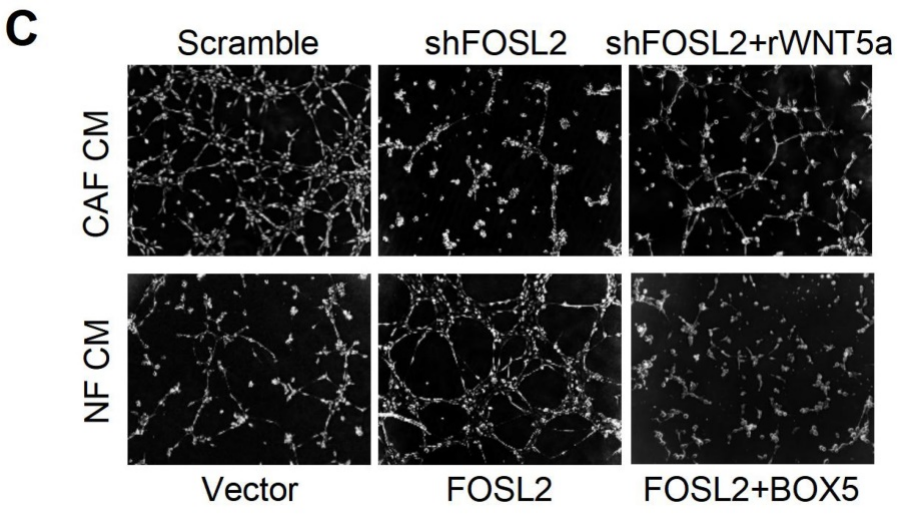
Corrected image for original Figure 6C

**Figure 2:**
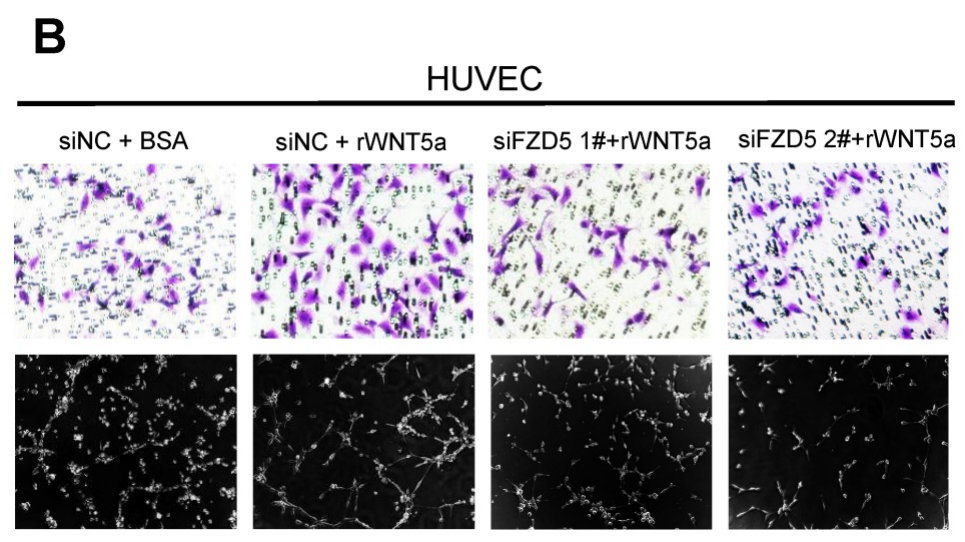
Corrected image for original Figure 7B

