# Supplementary material for: FOSL2 promotes VEGF-independent angiogenesis by transcriptionnally activating Wnt5a in breast cancer-associated fibroblasts
Source: Theranostics. 2021 Mar 5;11(10):4975–91. doi: 10.7150/thno.55074 (PMC7978317; doi:10.7150/thno.55074)
Supplement: Supplementary file 1 — Supplementary figures and tables. [file thnov11p4975s1.pdf]

Supplementary Figures and legends

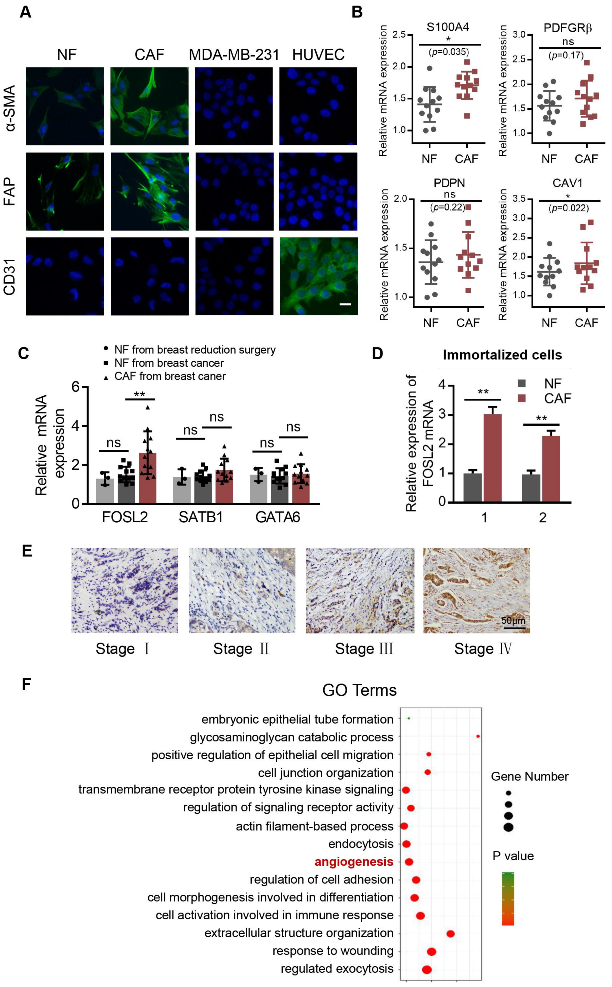

Figure S1. FOSL2 expression is upregulated and positively correlates with angiogenesis in breast cancer CAFs

(A) Immunophenotyping of established fibroblasts by immunofluorescence. In addition to CAFs and NFs, MDA-MB-231 cells, a breast cancer cell line, and HUVECs, an endothelial cell line, were used as controls (scale bar, 25 μm). (B) The primary fibroblasts were analyzed for other conventional fibroblast markers of PDGFRβ, S100A4, PDPN, and CAV1. Error bar shows median and interquartile range. (C) qRT-PCR to confirm the altered expression of FOSL2 in primary fibroblasts isolated from breast reduction surgery and breast tumors. (D) Expression levels of FOSL2 in immortalized fibroblasts. (E) Representative images of FOSL2 expression in breast tumor tissues were detected by ISH assays (scale bar, 50 μm). (F) GO analysis of genes potentially bound by FOSL2 to the promoter using the JASPAR and Cistrome DB databases.

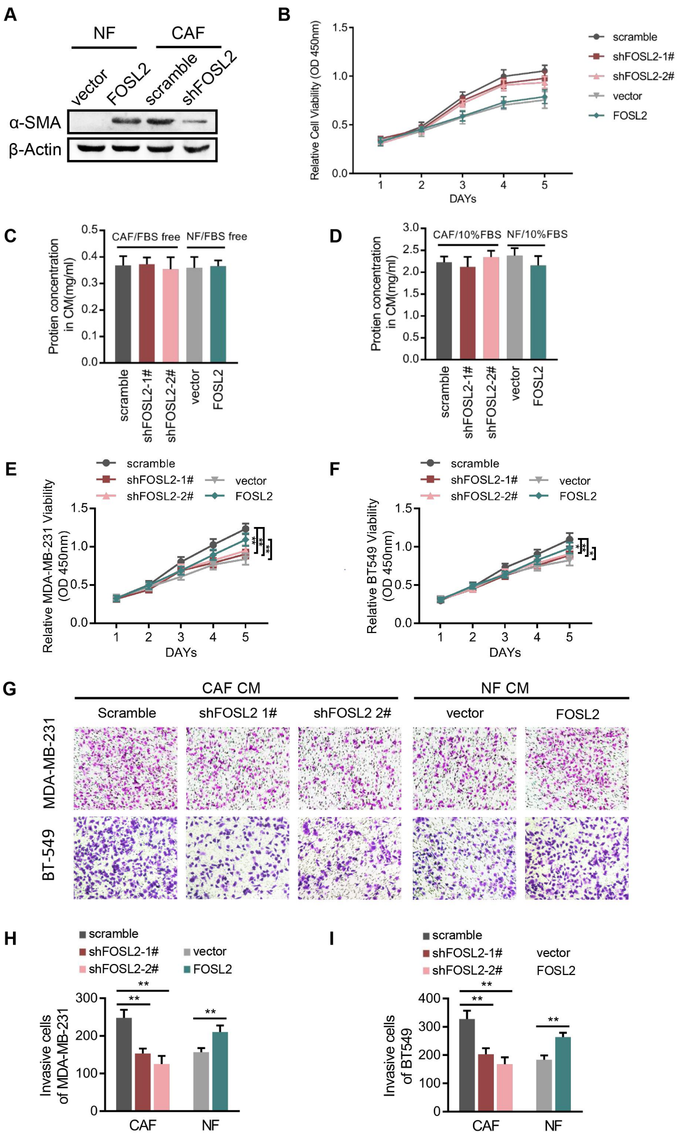

Figure S2. FOSL2 promotes CAF angiogenesis in vitro

(A) western blot analysis of α-SMA expression in the indicated NF or CAF cells. β-actin was used as a loading control. (B) Cell proliferation of stromal fibroblasts transfected with different types of vectors was tested by CCK-8 assay. (C, D) The indicated stromal fibroblasts were cultured in FBS-free medium or growth medium with 10% FBS for 20 hours. Protein concentrations in supernatant derived from fibroblasts cultured in FBS-free medium (C) or in growth medium (D) were tested by Bradford protein assay (p>0.05). (E-G) MDA-MB-231 and BT549 breast cancer cells were cocultured with CM from the indicated NFs or CAFs. Cell proliferation (E, F) or cell invasion (G, H, I) was tested by CCK-8 or transwell assays, respectively.

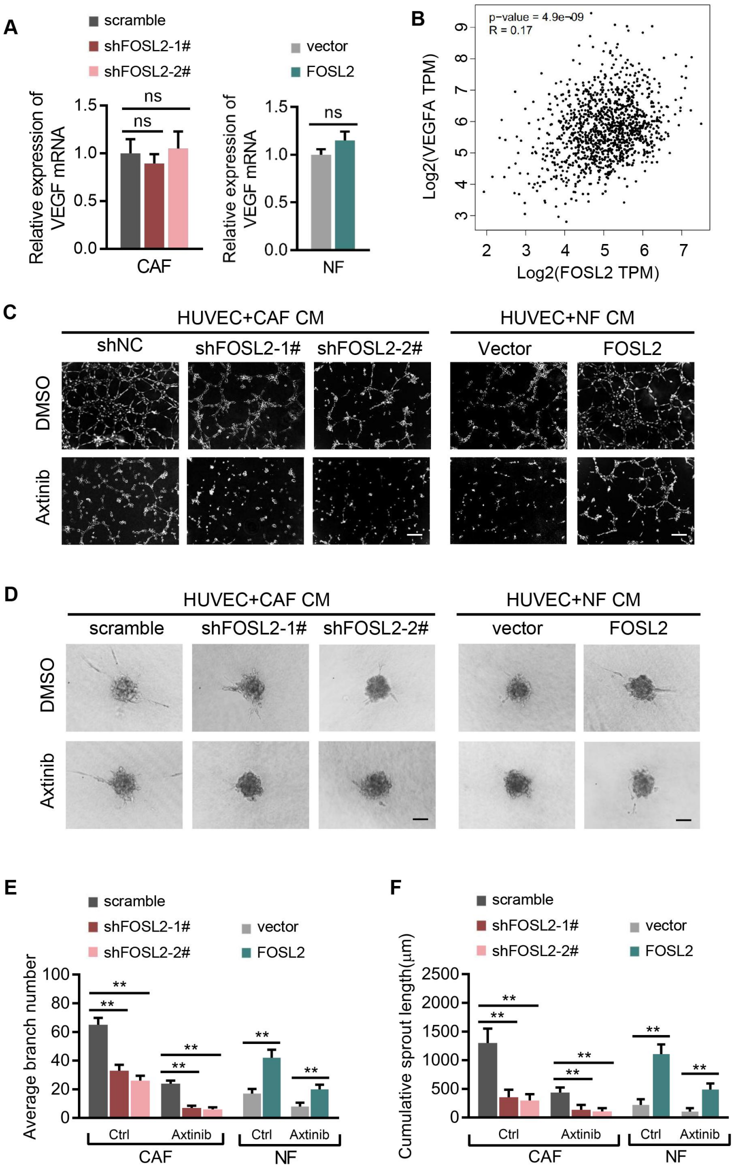

Figure S3. FOSL2 promotes CAF angiogenesis in a VEGF-independent manner. (A) VEGFA mRNA levels were determined by qRT-PCR and normalized to β-actin in the indicated stromal fibroblasts (***p*<0.01). (B) A positive correlation between FOSL2 and CD31 mRNA in samples from TCGA is shown (r=0.17, *p*<0.001). (C-F) CAFs with shRNA-1# or shRNA-2#, and FOSL2-overexpressing NFs and their control cells were cultured as described in the Materials section and treated with or without axitinib. Then, the collected CM was cocultured with HUVECs. Representative images of tube formation (C) and spheroid spouting (D) of HUVECs are shown. The average branch number of tubes (E) and cumulative sprout length of spheroid spouting (F) were calculated (scale bar, 100 μm).

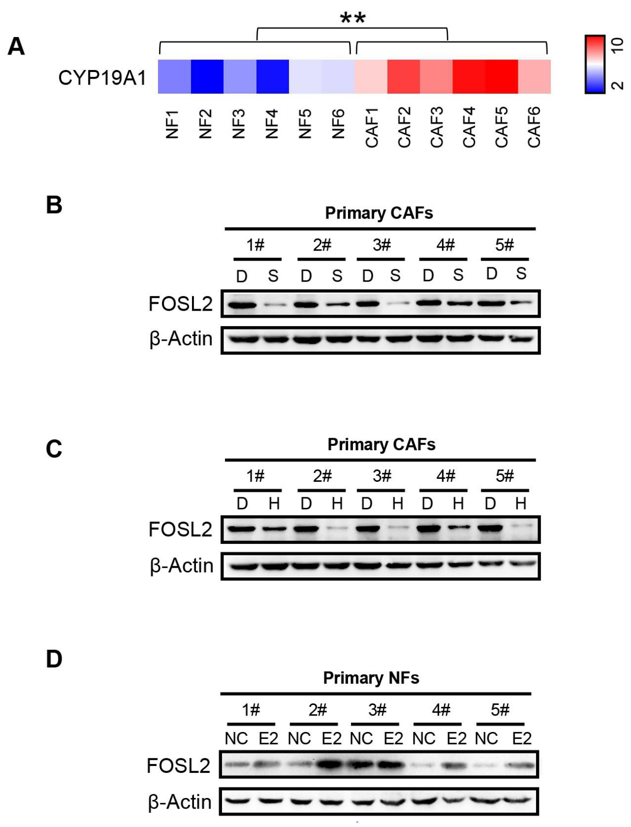

Figure S4. FOSL2 is regulated in primary CAFs by the estrogen/cAMP/PKA signaling axis

(A) Heat-Map of CYP19A1 in 6 paired breast CAFs and NFs detected by Agilent mRNA microarrays. (B-D) Protein levels of FOSL2 were detected in primary CAFs treated with or without SQ22536 (100 μM) (B) and H89 (5 μM) (C) or NFs treated with or without E2 (100 nM) (D) for 12 h (D: DMSO; S: SQ22536; H: H89).

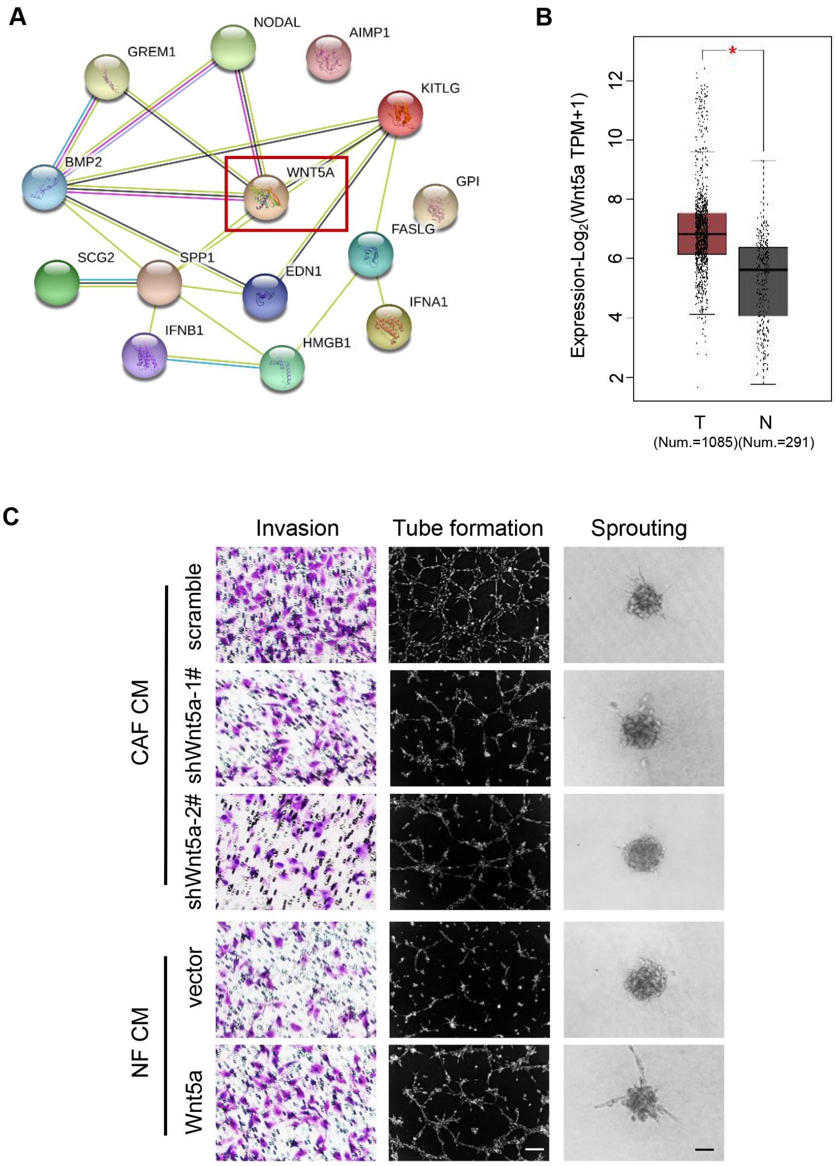

Figure S5. FOSL2 transcriptionally regulates Wnt5a expression and is involved in the FOSL2-mediated promotion of angiogenesis in CAFs

(A) Protein network analysis of the potential transcriptional target of FOSL2. (B) Relative expression of Wnt5a in breast tumor tissues (T) and normal tissues (N) was analyzed using TCGA data. (C) Representative images of HUVEC recruitment (left panel), HUVEC tube formation (middle panel), and HUVEC spheroid spouting (right panel) using conditioned media (CMs) from the indicated stromal fibroblasts are shown (scale bar, 100 μm).

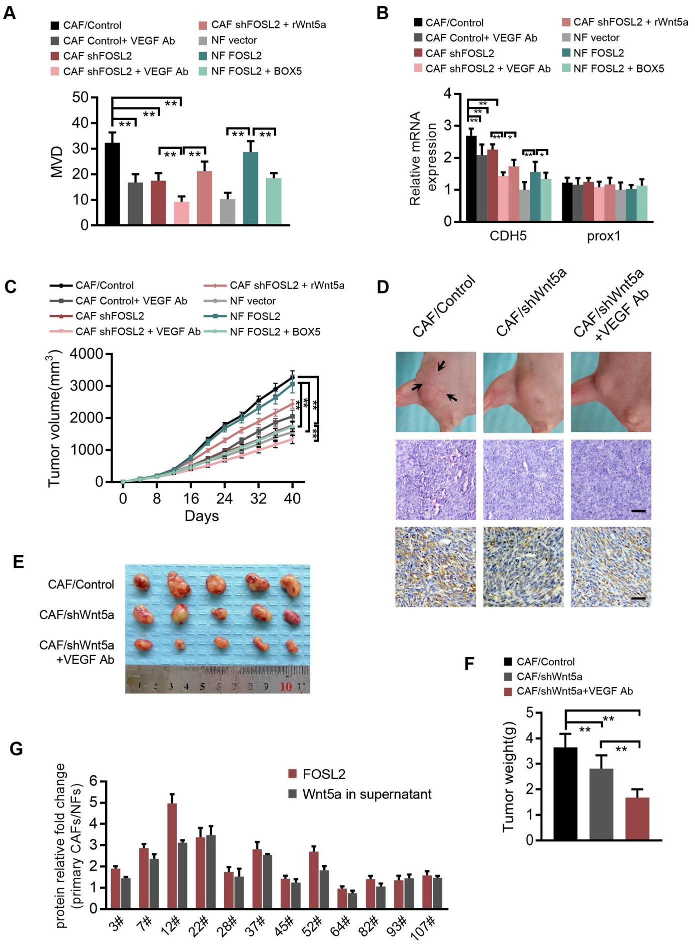

Figure S6. Tumor blood vessels and tumor weights measured in tumor-burden mice

(A) Analysis of the microvessel density in the indicated tumor group. (B) mRNA expression levels of VE-cadherin (CDH5) and prox1 in tumor samples. (C) The curves of tumor growth in mice. (D) Representative images of blood vessels on the tumor surface (labeled with black arrow) of mice injected with the mixture of MDA-MB-231 and CAF/shWnt5a under treatment with or without VEGF Ab. The blood vessel structures in the H&E and IHC staining of CD31 in tumors of each group of mice are shown (scale bar, 50 μm). (E-F) Representative tumor size (E) and tumor weight (F) for each group of xenograft mice are shown (**p<0.01). (G) Twelve pairs of primary CAFs and NFs were isolated from the matched noncancerous and cancerous tissues of breast cancer. The relative fold changes (CAFs vs NFs) of FOSL2 levels and secreted Wnt5a in the corresponding supernatant of stromal fibroblasts are shown, as determined by analysis of the gray level using ImageJ.

Table S1

The sequences of shRNA and small interfering RNA

Gene names	Sequence
Scramble	TTCTCCGAACGTGTCACGT
shFOSL2-1#	GGATTATCCCGGGAACTTT
shFOSL2-2#	CCACAGUGATCACCTCCAT
shWnt5a-1#	CTAGTGGCTTTGGCCATAT
shWnt5a-2#	CAAAGAATGCCAGTATCAA
siCYP19A1-1#	CGTTACACTTCTGAGACGA
siCYP19A1-2#	CCTAATGTTGAAGAGGCAA
siFZD5-1#	CATGATCCGCATCGGCATC
siFZD5-2#	ACATGGAACGCTTCCGCTA

Table S2

Primers for qRT-PCR

Gene names		Primers
FOSL2	FW<br>REV	5’-GTCACTCCGGGCACCTCGAAC-3’<br>5’- TTGGTCCCCGCTGCTACTGCT-3’
SATB1	FW<br>REV	5’-CTCGACCTTCCCAAGTACACC-3’<br>5’-ATATTCTGCCACATCGACCTC-3’
GATA6	FW<br>REV	5’-CCATGACTCCAACTTCCACCTC-3’<br>5’-GAGCCCATCTTGACCCGAAT-3’
Wnt5a	FW<br>REV	5’-ATTCTTGGTGGTCGCTAGGT-3’<br>5’-TGTACTGCATGTGGTCCTGA-3’
CYP19A1	FW<br>REV	5’-GGGCACATCCTCAATACCAG-3’<br>5’-CAGAAGGGTCAACACGTCCA-3’
FZD5	FW<br>REV	5’-ACACGCCCATCTGTCTGCC-3’<br>5’-CCTCGCTGCGGTTGTAATCC-3’
Wnt5a- CHIP(E1)	FW<br>REV	5’-TTTGCTTCTTTACCCACA-3’<br>5’-AGATGCAGTCAAACCTCC-3’
Wnt5a- CHIP(E2)	FW<br>REV	5’-AAGGACTTAGGTGGATAGA-3’<br>5’-ACAACACTGGCATCAAAT-3’
Wnt5a- CHIP(E3)	FW<br>REV	5’-TGCTTAGGGCAGGTATTG-3’<br>5’-GTCAGTGATGAAGCCACC-3’
S100A4	FW<br>REV	5’-GGACAGCAACAGGGACAA-3’<br>5’-TTCCTGGGCTGCTTATCT-3’
PDGFR β	FW<br>REV	5’-GTTCAAAGACAACCGCACCC-3’<br>5’-CAGCTCTGACACATACCGGG-3’
PDPN	FW<br>REV	5’-ATCACAAGCAGGAAGTCG-3’<br>5’-TCTGAGGGAAGTGGAGGG-3’
CAV1	FW<br>REV	5’-TGTCCGCTTCTGCTATCT-3’<br>5’-AAACATTCCTGGCTTCTC-3’
CDH5	FW<br>REV	5’-GACCGCCGTCTAACTCAA-3’<br>5’-CAGGCAGATAGGCACCAG-3’
Prox1	FW<br>REV	5’-ACCACGAGTCTGAGGACC-3’<br>5’-TTTATGAGCGACAAGGAG-3’
β-Actin	FW<br>REV	5’-TGACGTGGACATCCGCAAAG-3’<br>5’-CTGGAAGGTGGACAGCGAGG-3’

Table S3. Correlation between the expression of FOSL2 in CAFs and breast carcinoma characteristics and microvessel density

	FOSL2 expression in stroma				
	Low (48 cases)	High (69 cases)	Unpaired t	Chi-square	*p* value
Age (mean±S.D.)	58.2±10.6	56.7±10.4	0.761		0.4480
Tumor size					
＜2 cm	18	28		0.133	0.9357
2-5 cm	26	36			
＞5 cm	4	5			
Staging					
Ⅰ	15	10		7.894	0.0482*
Ⅱ	23	30			
Ⅲ	9	24			
Ⅳ	1	5			
Histological grade					
1	13	11		2.196	0.3335
2	29	49			
3	6	9			
Microvessel counts per field (CD31 stain)	20.9±4.8	24.7±4.5	4.371		＜0.0001**

$*p < 0.05$, $**p < 0.01$
